# Cerebellar activity in hemi-parkinsonian rats during volitional gait and freezing

**DOI:** 10.1093/braincomms/fcae246

**Published:** 2024-10-25

**Authors:** Valerie DeAngelo, Arianna Gehan, Siya Paliwal, Katherine Ho, Justin D Hilliard, Chia-Han Chiang, Jonathan Viventi, George C McConnell

**Affiliations:** Department of Biomedical Engineering, Stevens Institute of Technology, Hoboken, NJ 07030, USA; Department of Biomedical Engineering, Stevens Institute of Technology, Hoboken, NJ 07030, USA; Department of Biomedical Engineering, Stevens Institute of Technology, Hoboken, NJ 07030, USA; Department of Biomedical Engineering, Stevens Institute of Technology, Hoboken, NJ 07030, USA; Department of Neurosurgery, University of Florida, Gainesville, FL 32611, USA; Department of Biomedical Engineering, Duke University, Durham, NC 27708, USA; Department of Biomedical Engineering, Duke University, Durham, NC 27708, USA; Department of Neurosurgery, Duke School of Medicine, Durham, NC 27710, USA; Department of Neurobiology, Duke School of Medicine, Durham, NC 27710, USA; Duke Comprehensive Epilepsy Center, Duke School of Medicine, Durham, NC 27710, USA; Department of Biomedical Engineering, Stevens Institute of Technology, Hoboken, NJ 07030, USA

**Keywords:** cerebellar vermis, 6-OHDA, Parkinson’s disease, μECoG

## Abstract

Parkinson’s disease is a neurodegenerative disease characterized by gait dysfunction in the advanced stages of the disease. The unilateral 6-hydroxydopamine toxin-induced model is the most studied animal model of Parkinson’s disease, which reproduces gait dysfunction after >68% dopamine loss in the substantia nigra pars compacta. The extent to which the neural activity in hemi-parkinsonian rats correlates to gait dysfunction and dopaminergic cell loss is not clear. In this article, we report the effects of unilateral dopamine depletion on cerebellar vermis activity using micro-electrocorticography during walking and freezing on a runway. Gait and neural activity were measured in 6-hydroxydopamine- and sham-lesioned rats aged between 4 and 5 months at 14, 21 and 28 days after infusion of 6-hydroxydopamine or control vehicle into the medial forebrain bundle (*n* = 20). Gait deficits in 6-hydroxydopamine rats were different from sham rats at 14 days (*P* < 0.05). Gait deficits in 6-hydroxydopamine rats improved at 21 and 28 days except for run speed, which decreased at 28 days (*P* = 0.018). No differences in gait deficits were observed in sham-lesioned rats at any time points. Hemi-parkinsonian rats showed hyperactivity in the cerebellar vermis at 21 days (*P* < 0.05), but not at 14 and 28 days, and the activity was reduced during freezing epochs in Lobules VIa, VIb and VIc (*P* < 0.05). These results suggest that dopaminergic cell loss causes pathological cerebellar activity at 21 days post-lesion and suggest that compensatory mechanisms from the intact hemisphere contribute to normalized cerebellar activity at 28 days. The decrease in cerebellar oscillatory activity during freezing may be indicative of neurological changes during freezing of gait in patients with Parkinson’s disease making this region a potential location for biomarker detection. Although the unilateral 6-hydroxydopamine model presents gait deficits that parallel clinical presentations of Parkinson’s disease, further studies in animal models of bilateral dopamine loss are needed to understand the role of the cerebellar vermis in Parkinson’s disease.

## Teaser

DeAngelo *et al*. studied cerebellar neural activity during gait in a neurotoxin-induced rat model of Parkinson’s disease. Dopamine loss was correlated with hyperactivity in the cerebellar vermis during walking, which was reduced during freezing. Our results suggest the vermis as a biomarker for therapies to treat Parkinson’s disease.

## Graphical Abstract

**Graphical Abstract:**
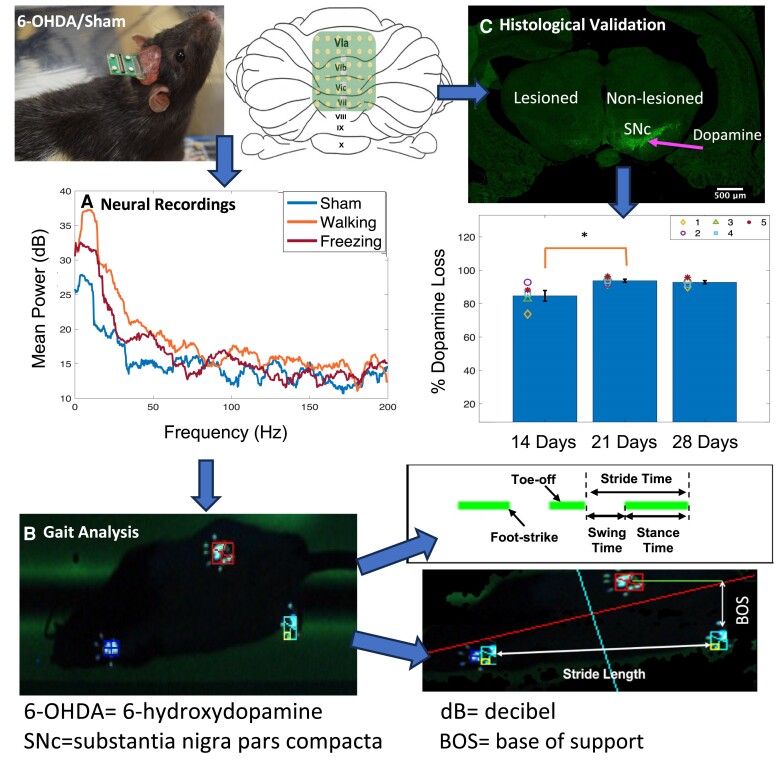

## Introduction

Parkinson’s disease is a progressive neurodegenerative disorder that causes symptoms of gait dysfunction, including freezing of gait (FOG). Although the exact mechanism is unclear, gait deficits typically appear with substantia pars compacta (SNc) dopamine (DA) loss exceeding 40% and become more pronounced after 68% loss.^1-6^ Symptoms present as shuffled gait, decreased stride length and decreased velocity.^6^ FOG is the most difficult symptom to treat and is defined as the episodic absence or marked reduction of forward progression of gait despite the intention to walk.^7,8^

Studies have found that symptoms of parkinsonian gait are consistently replicated in the unilateral 6-hydroxydopamine (6-OHDA) model of Parkinson’s disease following 68% DA loss.^6,8-10^ Sufficient DA loss for gait dysfunction occurs as early as 7 days post-6-OHDA infusion into the medial forebrain bundle (MFB) and the gait dysfunction is dose dependent.^11^ Zhou *et al*.^12^ suggested that an increased duration of stance time may indicate FOG and gait hesitation. The similarities between the acute 6-OHDA gait deficits and clinical presentations in advanced Parkinson’s disease support the assertions that this model has good face validity to further our understanding of the circuit mechanisms involved in parkinsonian gait.^13^

The cerebellum—a brain region important for motor planning, control and execution—is known to exhibit aberrant activity in patients with Parkinson’s disease.^14-19^ During normal gait, movement is initiated based on volitional or emotional processes followed by automatic processes which generate limb movements and regulate postural muscle tone.^20-22^ The cerebellum acts on the cerebral cortex to regulate volitional processes and works in conjunction with the cerebral cortex and basal ganglia (BG) to control motor planning, control and execution that is driven by emotion, motivation and cognition.^23,24^ Further, sensory information is modulated by cholinergic projections from the pedunculopontine nucleus (PPN) to the cerebellum to provide feedback important for posture and gait.^25^

Cerebellar vermis (CBLv) lobules are associated with varying cognitive, emotional and motor function. Motor areas of the cerebellum are predominately localized to vermis Lobules VI and VII, which are involved in the regulation of motor dexterity, coordination and complex movements. Cerebellar cognitive and limbic regions, located in Lobule VII, are involved in cognition, emotion and motor planning.^26,27^

CBLv contributions to gait dysfunction in Parkinson’s disease have been linked to degeneration in the PPN and BG.^1,28,29^ Direct connections between the CBLv, BG and PPN provide pathways for pathological activity to alter cerebellar function in Parkinson’s disease. To our knowledge, the current understanding of CBLv changes in Parkinson’s disease is limited to results from imaging studies, which measure brain wave activity indirectly by coupling activity to increases in blood flow^30^ and EEG recordings, which directly measure brain wave activity in the delta, theta, alpha, beta and gamma frequency bands to detect abnormalities but have limited spatial resolution.^31^ Generally, delta waves have been related to behavioural inhibition and cognitive processes;^32^ theta waves occur during movement navigation;^33^ alpha waves are activated during wakefulness and are associated with sensory perception and cognitive inhibition; beta waves are associated with movement, complex tasks and decision making; and gamma waves are linked to states of high attention.^34^

Hanakawa *et al*. found that the CBLv is hyperactive in patients with Parkinson’s disease experiencing gait dysfunction. Most studies that have measured CBLv hyperactivity in Parkinson’s disease were performed indirectly using imaging methods. These methods typically measure activity before, after or in the absence of gait.^14-19^

A more recent electrophysiological study by Bosch *et al*.^31^ using EEG found CBLv activity was attenuated during cognitive processing and lower limb performance in Parkinson’s disease subjects compared with healthy controls. To our knowledge, this is one of the few studies to measure the electrophysiology of the CBLv.

We measured the oscillatory activity of CBLv Lobules VIa, VIb, VIc and VII in 6-OHDA rats aged between 4 and 5 months using micro-electrocorticographic (μECoG) arrays.^35^ The μECoG arrays enabled high spatial resolution electrophysiological recordings from CBLv during freezing and continuous gait and the ability to discriminate individual lobules with the CBLv.^35^ Gait and power spectral analysis performed 14, 21 and 28 days after the 6-OHDA infusion allowed us to directly measure the effects of DA depletion in the SNc on gait and cerebellar activity. Our results provide insight into the face validity of the unilateral 6-OHDA model for gait dysfunction in Parkinson’s disease and the underlying neural activity in the CBLv.

## Material and methods

### Animals and housing

Adult male Long–Evans hooded rats (*n* = 25) were purchased from Charles River Laboratories. All rats were between 4 and 5 months of age and were singly housed (post-surgery) in standard cages with free access to food and water. Study protocols were reviewed and approved by the Stevens Institute of Technology Institutional Animal Care and Use Committee.

### Experimental design

6-OHDA infusion in the MFB causes degeneration of dopaminergic (DAergic) neurons in the SNc. To measure the effect of DA loss on cerebellar activity, three groups of rats were lesioned with 6-OHDA: Group A (*n* = 7), Group B (*n* = 7) and 6-OHDA (*n* = 6). Groups A and B rats were perfused 14 and 21 days post-lesion, respectively, to measure the time effect of 6-OHDA on DA loss (these rats did not undergo behavioural testing). Rats in the 6-OHDA group were implanted with μECoG array to longitudinally record CBLv activity during gait at 14, 21 and 28 days post-lesion/implantation. The control group (*n* = 5), labelled sham, was infused with sterile saline and implanted with a μECoG array. 6-OHDA and sham rats were perfused at 28 days post-surgery. Neural and gait analyses were performed unblinded while immunohistochemistry and cell counting were performed blinded.

### Unilateral 6-OHDA lesion and chronic μECoG implantation

Sterile stereotaxic surgery was conducted under 4% sevoflurane anaesthesia using coordinates from a rat brain atlas.^36^ Rats were placed in a stereotaxic frame and a craniotomy was made over the CBLv (AP −9.5 to −14.5 mm, ML ±4 mm) and the MFB (AP −1.8 mm, ML + 2.0 mm, DV −8.6 mm relative to bregma). Eight stainless steel screws were anchored to the skull, including two screws placed over the visual cortex, which served as reference and ground. Thirty minutes prior to infusion rats were pre-treated with intraperitoneal (IP) injections of 50 mg/kg pargyline (Sigma–Aldrich) and 5 mg/kg desipramine (Sigma–Aldrich) to inhibit monoamine oxidase and protect non-adrenergic neurons, respectively.^37^

**Figure 1:**
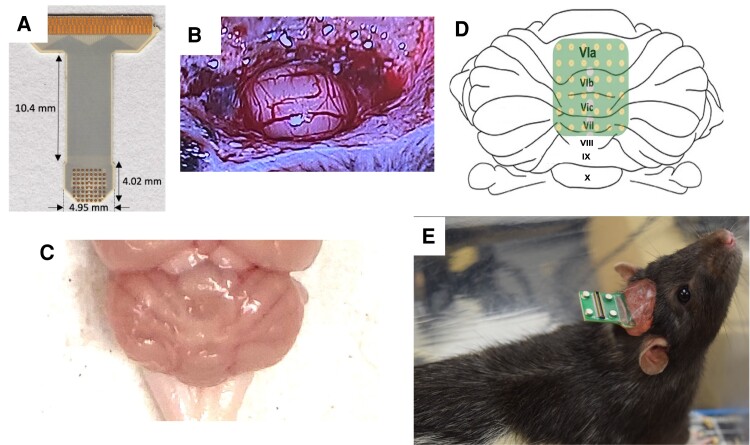
**Chronic μECoG implantation.** (**A**) μECoG array arranged in an 8 × 8 grid with 230 μm diameter spaced 420 μm apart. (**B**) Craniotomy created over CBLv (**C**) Lobules VIa through VII. (**D**) Approximate location of the μECoG array when chronically implanted. (**E**) Finished head cap. μECoG, micro-electrocorticography.

Immediately before infusion, 6-OHDA hydrobromide (Tocris Bioscience) was dissolved in ice-cold 0.02% ascorbic acid dissolved in saline to a concentration of 4 μg/μl. Four microlitres of 6-OHDA were infused into the MFB of the right hemisphere at a rate of 1 μl/min for a total of 16 μg. Sham rats did not receive desipramine/pargyline and were infused with 4 μl of sterile saline in lieu of 6-OHDA. Immediately following infusion, a μECoG array, arranged in a 8 × 8 grid of 61 total electrodes with 230 μm diameter electrodes spaced 420 μm (Fig. 1A), was implanted over CBLv Lobules VIa (AP −11.205 to −11.855 mm, ML ±1.5 mm), VIb (AP −12.045 to −12.7 mm, ML ±1.5 mm), VIc (AP −12.88 to −13.21 mm, ML ±1.3 mm) and VII (AP −13.4 to −14.05 mm, ML ±1.5 mm) in 6-OHDA and sham rats (Fig. 1B–D). Dental acrylic was used to secure the μECoG array to stainless steel screws (Fig. 1E). Sterile Covidien Vaseline petroleum jelly was placed over the craniotomy to create a barrier between the dental acrylic head cap and the brain surface. Electrode location was confirmed after perfusion using a Vernier calliper.

### Methamphetamine-induced circling

Seven days post-lesion, rats from the 6-OHDA group, Group A and Group B were administered 1.6 mg/kg of methamphetamine through IP injection and placed in a dark circling chamber for 1.5 h. An infrared camera captured the rat’s activity. The lesion was considered successful if the rat circled at a minimum average rate of three turns per minute indicative of >80% DA loss as confirmed by TH staining.^37^ Rats that did not have a successful lesion based on this criteria were excluded from the remainder of this study.

### Gait analysis

Gait assessment was performed on the 6-OHDA (*n* = 5) and sham (*n* = 5) rats using a runway system for gait analysis (CSI-G-RWY; CleverSys Inc., Reston, VA, USA). As the rat voluntarily moved across the runway, comprised of a long side-lit glass plate, each footprint was lit up and recorded by a camera mounted under the glass. Prior to assessment, each rat was trained daily for three consecutive runs until they walked across the apparatus without hesitation. Each rat was then evaluated at 14, 21 and 28 days post-lesion. Data from three consecutive runs were averaged for each rat at each time point. Each video was analysed using the gait analysis software (GaitScan; CleverSys Inc.). After 5 min, if the 6-OHDA subject would not walk on the runway, they were placed back in their home cage for 3 min and an attempt was made again.

Analysis was performed for periods of walking (continuous gait consisting of at least three consecutive steps) and freezing immediately following continuous gait. Freezing events were defined as cessation of continuous gait with at least three paws in contact with the glass for >500 ms. Analysis was performed on swing time (in ms; time in which paw is in the air), stance time (in ms; time in which all paws are detected on the glass), stride time (in ms; stance time plus swing time), stride length (in mm; distance the paw traversed from the start of the previous stance to the beginning of the next stance) and run speed (in mm/s; instantaneous speed over a running distance).^38^

### Neural recordings

**Figure 2:**
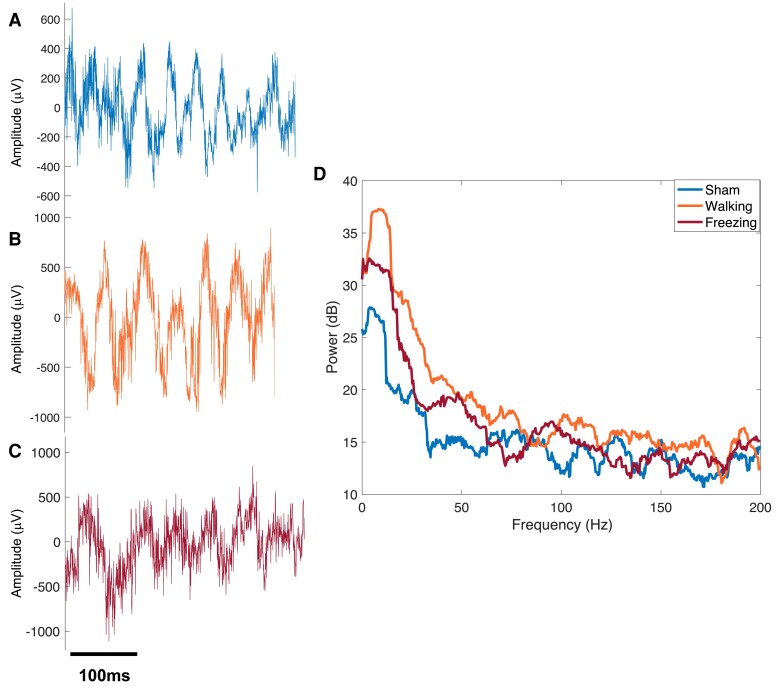
**Sample neural recordings.** (**A**) Sample LFPs recorded from CBLv Lobule VIa of sham control during walking and 6-OHDA during (**B**) walking and (**C**) freezing. (**D**) Sample power spectra [decibels (dB)] calculated from LFPs measured in sham, 6-OHDA during walking and 6-OHDA during a bout of freezing during volitional gait.

Local field potential (LFP) recordings were taken during volitional gait on the runway using the Open Ephys (open-source electrophysiology) system (Fig. 2A and B).^38^ The runway was modified to synchronize LFP recordings with gait. Two beam break sensors were used in conjunction with an Arduino to control the timing of the LFP recording period. As the rat left its start box and entered the field of view, the first sensor was broken, a TTL pulse was sent to Open Ephys to start recording and an LED was turned on. LFPs were continuously recorded as the rat traversed the runway. After the rat exited the runway and entered the exit box, a second sensor was broken sending a TTL pulse to stop the recordings and turn off the LED. The LED served as a visual indicator for the start and end of LFP recordings during gait analysis. Data from three consecutive runs were stored for each rat for further analysis.

### Neural data analysis

Continuous LFP recordings were exported and analysed using MATLAB (Mathworks, Natick, MA, USA). First, LFP timing was synchronized with the LED ON and OFF video frames to crop recordings based on walking and freezing for 6-OHDA rats as sham rats did not freeze on the runway. Next, LFP recordings were separated into epochs of walking and freezing based on the runway videos. LFP recordings during the first three-fourth of the length of the runway were analysed, because most rats (sham and 6-OHDA) stopped at the end of the runway prior to exiting into their home cage. Multitaper methods of spectral estimation were used to quantify changes in CBLv oscillatory activity between 6-OHDA (Fig. 2B and C) (walking and freezing) and sham rats (Fig. 2A; walking) in each lobule (Chronux version 2.00; Fig. 2D). The LFP spectrum was estimated on a 2-s window with 10 Hz resolution using 19 Slepian data tapers.^37^ The mean power in dB was measured within the following frequency bands: theta (4–8 Hz), alpha (8–13 Hz), low beta (13–20 Hz), high beta (21–30 Hz), low gamma (30–55 Hz), high gamma (65–80 Hz) and fast frequency (80–200 Hz). Frequencies between 55 and 65 Hz were omitted to reduce effects of 60 Hz noise.^39^

### Immunohistochemistry

Fourteen-, 21 and 28 days post-lesion rats were deeply anaesthetized and transcardially perfused with phosphate-buffered saline (PBS) followed by 10% formalin. The brain was removed and post-fixed overnight (4°C) in formalin, then placed in a 30% sucrose (4°C) solution until it sank. A green tissue marking dye was applied to the left-posterior hemisphere to demarcate the orientation of the brain sections. The brains were cryoprotected with Tissue-Tek optimal cutting temperature compound, and 40 μm serial coronal sections were cut using a cryostat (CryoStar NX50) equally spaced through the substantia nigra pars compacta (SNc). Immunohistochemistry was performed in the SNc with anti-tyrosine hydroxylase (TH) antibody to measure DAergic neuron loss.^38^ After three rinses in PBS, sections were blocked for 1 h at 4°C in blocking solution containing PBS, normal goat serum (NGS) and 10% Triton-X. The sections were then incubated in anti-TH monoclonal rabbit IgG primary antibody (1:2000 overnight at 4°C; Sigma–Aldrich) in solution with PBS and NGS. Next, the sections were incubated with goat anti-rabbit IgG Alexa 488 secondary antibody (1:500 for 2 h at 4°C; Invitrogen by Thermo Fisher Scientific) in solution with PBS, NGS and 10% Triton-X. Sections were mounted in FluoroMount-G and imaged using the Keyence BZ-X700 series microscope (Supplementary Fig. 1).

### Cell counting

Automated cell counting was performed using ImageJ.^38,40,41^ DAergic degeneration was determined in the SNc by counting the number of TH-positive cells of each rat (Supplementary Fig. 1). Each 10× image was converted from RGB to 16-bit greyscale. The threshold was set to a pixel intensity of 30 (arbitrary units) and cells were analysed in a region of interest restricted to a 705.9 mm (*w*) × 564.7 mm (*h*) rectangle on either side of the midline in the SNc. The percentage of DA loss of the lesioned side normalized to the non-lesioned side was measured using the following equation:^37^


(1) $$\begin{array}{rl} & \text{\%}\;\;\mathrm{Dopaminergic}\;\mathrm{loss}\;=\\ & \left|\left(1-\;\frac{\text{\#of}\;{\mathrm{TH}}^{+\;}\;\mathrm{cells}\;\mathrm{on}\;\mathrm{lesioned}\;\mathrm{side}}{\text{\#of}\;{\mathrm{TH}}^{+\;}\;\mathrm{cells}\;\mathrm{on}\;\mathrm{non}\text{-}\mathrm{lesioned}\;\mathrm{side}}\right)\times 100\right| \end{array}$$

### Statistical analysis

Statistical analysis was performed using IBM SPSS Statistics for Mac (IBM Corp., Armonk, NY, USA). Means were compared using a two-way mixed ANOVA with time and type as factors for gait and LFP power comparisons between 6-OHDA and sham rats. For significant interactions, univariate analysis and one-way repeated measures ANOVA were used to determine the significance between types at each time point and between time points for each type, respectively. The percent DA loss of the Group A, Group B, 6-OHDA (28 days) and sham rats (control) was compared using a one-way ANOVA. A Sidak *post hoc* test was used for all analyses. The results were considered statistically significant at *P* ≤ 0.05.

## Results

### Methamphetamine-induced circling

A total of 20 rats were administered 6-OHDA in this study [Group A (*n* = 7), Group B (*n* = 7) and 6-OHDA (*n* = 6)]. Of these 20 rats, 5 lesions did not meet the minimum of 3 turns per minute over the period of 1.5 h post-6-OHDA injection and excluded from further analysis. Of the 15 rats that met the minimum circling rate condition 5 rats in Group A circled an average of 12.3 turns per minute, 5 rats in Group B an average of 12.5 turns per minute and 5 rats in 6-OHDA an average of 10.3 turns per minute.

### Gait analysis

Gait dysfunction was observed across all measurements between 6-OHDA (*n* = 5) and sham control rats (*n* = 4). Gait analysis for one of the sham control rats could not be performed due to data corruption of the video files. 6-OHDA rats had periods of freezing at the beginning, middle and end of the runway at each time point, while the sham rats only paused at the end when entering their home cage. This was removed from analysis, as it was not deemed as a freezing episode. Start hesitation was also observed at each time point. During multiple runs, 6-OHDA continuously circled at the start box, which resulted in no forward progression of gait. Placing the rat back in their home cage for 3 min and restarting the trial typically resulted in a successful run where the rat was able to traverse down the runway, allowing gait measurement.

**Figure 3:**
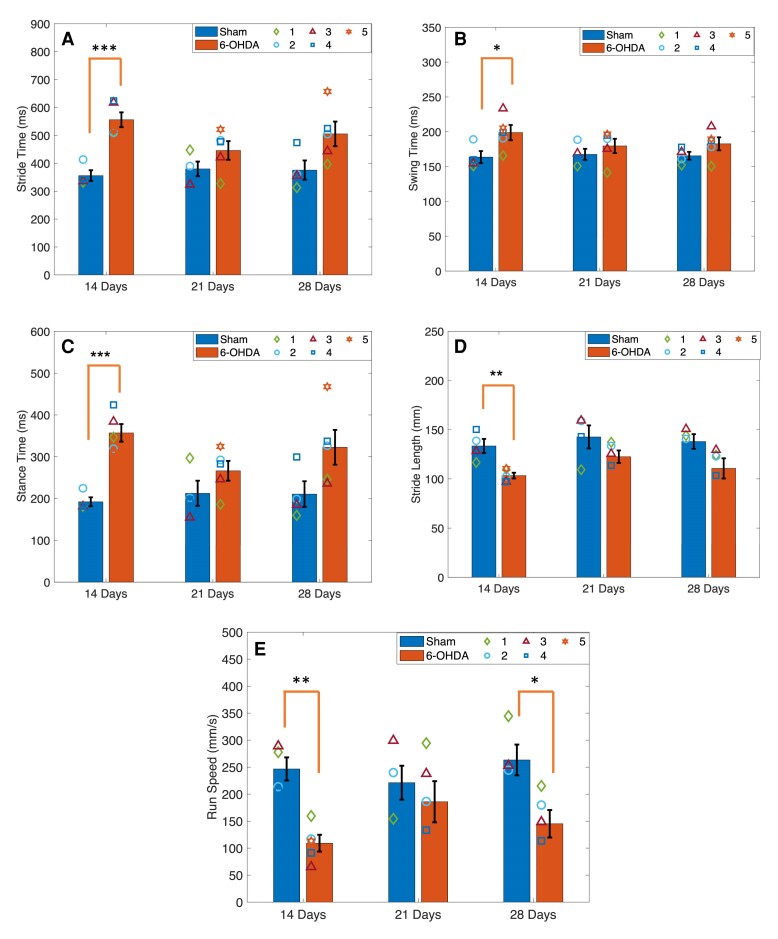
**Gait analysis.** (**A**) Stride time, (**B**) swing time and (**C**) stance time were measured in 6-OHDA-lesioned rats and sham rats at 14, 21 and 28 days post-lesion. Stride time, swing time and stance time were significantly greater in 6-OHDA-lesioned rats compared with sham at 14 days but not at 21 and 28 days. (**D**) Stride length was significantly greater in 6-OHDA rats at 14 days compared with sham controls. (**E**) 6-OHDA had a significantly slower run speed than sham controls at both 14 and 28 days, but not 21 days. Symbols represent the mean at each time point in 6-OHDA rats (*n* = 5) and sham control rats (*n* = 4). *P < 0.05, **P < 0.01 and ***P < 0.001. Bars indicate mean ± standard error of the mean (SEM). Comparisons were made using univariate statistical analysis.

Stride time, stance time and swing time were compared between 6-OHDA rats and sham controls. 6-OHDA rats experienced gait deficits when compared with age matched sham controls only at 14 days post-lesion (Supplementary Table 1). No statistically significant interaction between type and time was found for stance time [*F*(2,14) = 1.897, *P* = 0.187], swing time [*F*(2,14) = 2.218, *P* = 0.146] and stride time [*F*(2,14) = 2.507, *P* = 0.117]. Significant differences were found for the main effect of type for stride time [*F*(1,7) = 17.431, *P* = 0.004] and stance time [*F*(1,7) = 20.637, *P* = 0.003], but not swing time [*F*(1,7) = 3.462, *P* = 0.105]. 6-OHDA rats had greater stride time and stance time when compared with the sham control. Simple effects analysis revealed significant differences between 6-OHDA and sham at 14 days but not at 21 or 28 days for stride time [*F*(1,7) = 33.820, *P* < 0.001], stance time [*F*(1,7) = 41.366, *P* < 0.001] and swing time [*F*(1,7) = 5.820, *P* = 0.047]. No significant main effect of time was found across temporal gait measures. Overall, stance time, stride time and swing time were greater in 6-OHDA-lesioned rats than sham lesioned at all time points (Fig. 3).

There was no significant two-way interaction between type and time for stride length (Fig. 3D). The main effect of type was significant [*F*(1,7) = 11.705, *P* = 0.011], while the main effect of time was not. Simple main effects analysis found significant differences between 6-OHDA and sham rats only at 14 days [*F*(1,7) = 17.909, *P* = 0.004]. Stride length was decreased at all time points in 6-OHDA rats compared with sham.

There was no significant two-way interaction between type and time on run speed. The main effect for type was statistically significant [*F*(1,7) = 10.303, *P* = 0.015]. Simple effects showed a significant reduction in speed at 14 [*F*(1,7) = 28.476, *P* = 0.001] and 28 days [*F*(1,7) = 9.563, *P* = 0.018] in 6-OHDA compared with sham (Fig. 3E). No significant main effect was found for time.

### Mean power

LFP average logarithmic power was calculated in CBLv Lobules VIa, VIb, VIc and VII during periods of walking and freezing in the delta, theta, alpha, low-beta, high-beta, low-gamma, high-gamma and fast frequency bands. Activity during walking was compared between 6-OHDA and sham rats at 14, 21 and 28 days after surgery. Mean power during freezing was compared with mean power during periods of walking in 6-OHDA-lesioned rats at all time points. Vermis lobules of 6-OHDA rats were hyperactive at 14 and 21 days when compared with sham rats in all frequency bands in all lobules. This activity normalized to levels of the sham group at 28 days.

#### Walking

**Figure 4:**
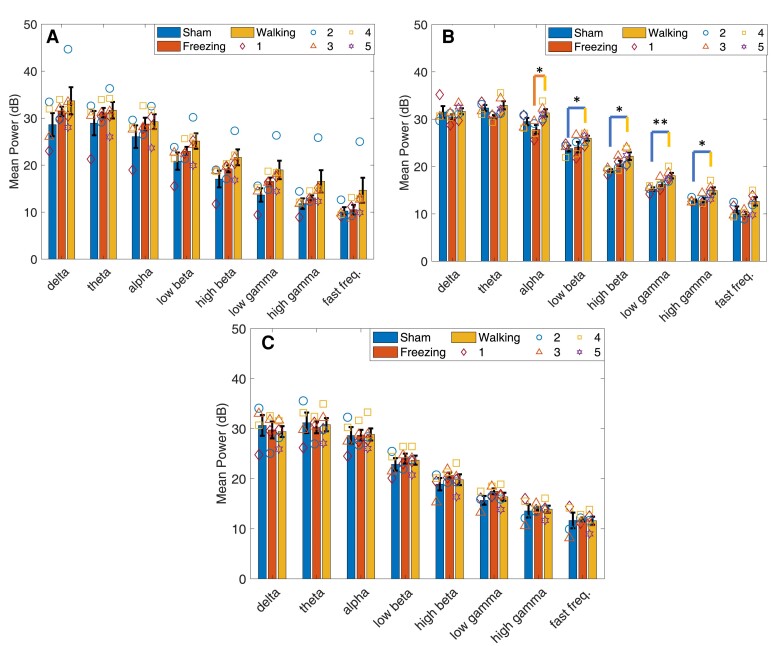
**Mean power calculated in Lobule VIa.** Cerebellar activity was measured in Lobule VIa of sham and 6-OHDA rats during walking and walking and freezing, respectively, at 14, 21 and 28 days post-lesion. (**A**) Mean power [decibels (dB)] measured in sham rats during walking and 6-OHDA rats during walking and freezing 14 days post-lesion. No significant differences were found between types. (**B**) Mean power measured in sham and 6-OHDA rats at 21 days post-lesion. Hyperactivity was measured in 6-OHDA rats during walking in the low-beta, high-beta, low-gamma and high-gamma frequency bands compared with sham. A significant difference was measured between walking and freezing in 6-OHDA rats in the alpha band (**C**). At 28 days no significant differences in power were measured. Symbols represent the mean at each time point in (*n* = 5) 6-OHDA rats and (*n* = 4) sham control rats. *P < 0.05; **P < 0.01. Bars indicate mean ± SEM. Comparisons were made using univariate statistical analysis.

No statistically significant interaction between type and time was found for any of the frequency bands in all lobules during walking. The Lobule VIa main effect type was significant in the high beta [*F*(1,7) = 6.277, *P* = 0.041] and low gamma [*F*(1,7) = 7.794, *P* = 0.027] bands (Fig. 4). Simple effects analysis revealed significant hyperactivity in 6-OHDA-lesioned rats at 21 days in the low-beta [*F*(1,7) = 7.2, *P* = 0.031], high-beta [*F*(1,7) = 10.204, *P* = 0.015], low-gamma [*F*(1,7) = 14.769, *P* = 0.006] and high-gamma [*F*(1,7) = 7.105, *P* = 0.032] bands compared with sham controls.

**Figure 5:**
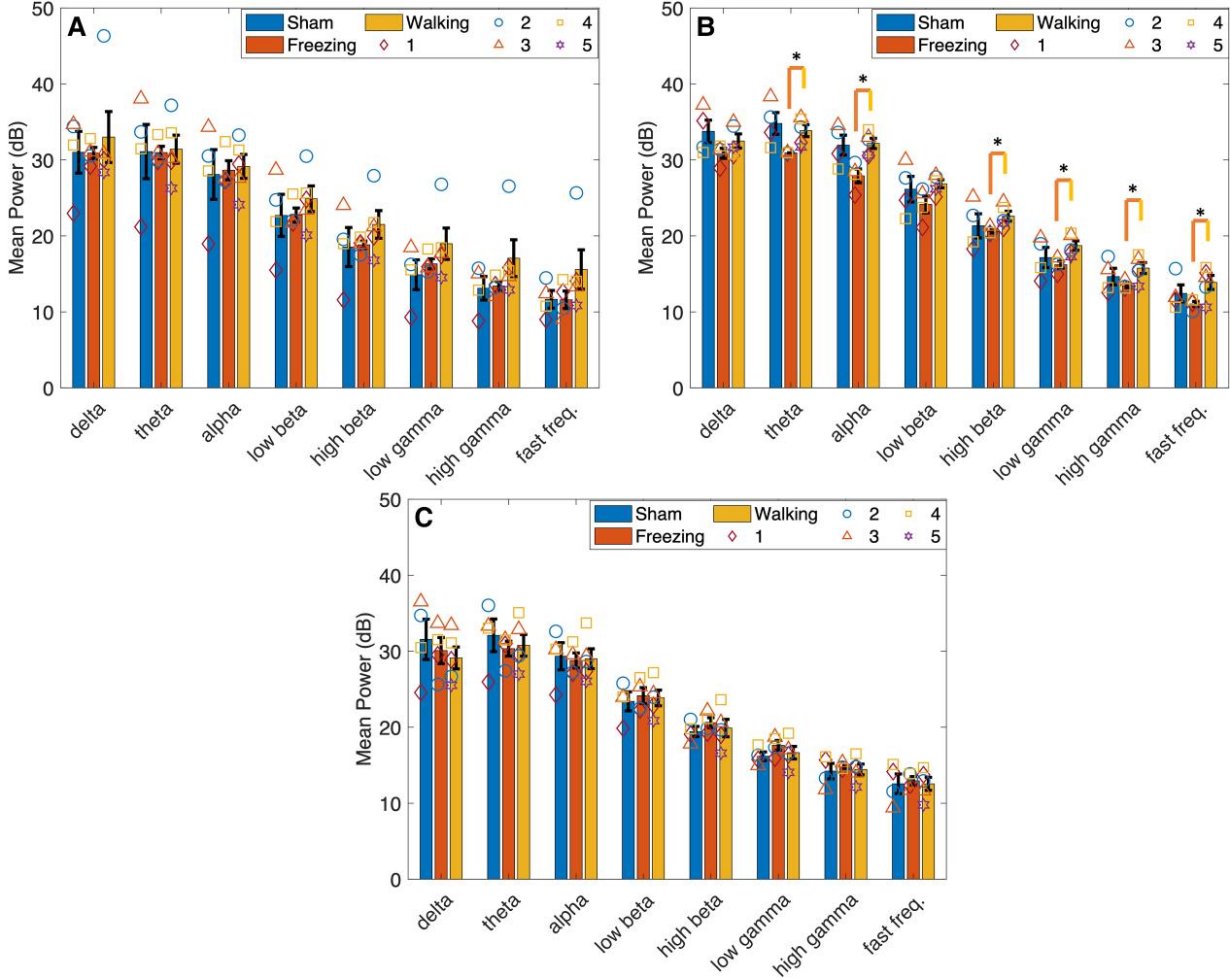
**Mean power calculated in Lobule VIb.** Cerebellar activity was measured in Lobule VIb of sham and 6-OHDA rats during walking and walking and freezing, respectively, at 14, 21 and 28 days post-lesion. (**A**) No significant differences were found in mean power [decibels (dB)] measured in sham rats during walking and 6-OHDA rats during walking and freezing 14 days post-lesion. (**B**) Mean power measured in sham and 6-OHDA rats at 21 days post-lesion. Hypoactivity was measured in 6-OHDA rats during freezing in the theta, alpha, high-beta, low- and high gamma, and fast frequency bands compared with walking. No significant differences were measured during walking between power in 6-OHDA and sham rats. (**C**) At 28 days no significant differences in power were measured. Power was significantly higher at 21 days post-lesion compared with 28 days in 6-OHDA rats in the alpha and low-gamma bands during walking. These associations can also be seen in Fig. 6C. Symbols represent the mean at each time point in (*n* = 5) 6-OHDA rats and (*n* = 4) sham control rats. *P < 0.05. Bars indicate mean ± SEM. Comparisons were made using univariate statistical analysis.

**Figure 6:**
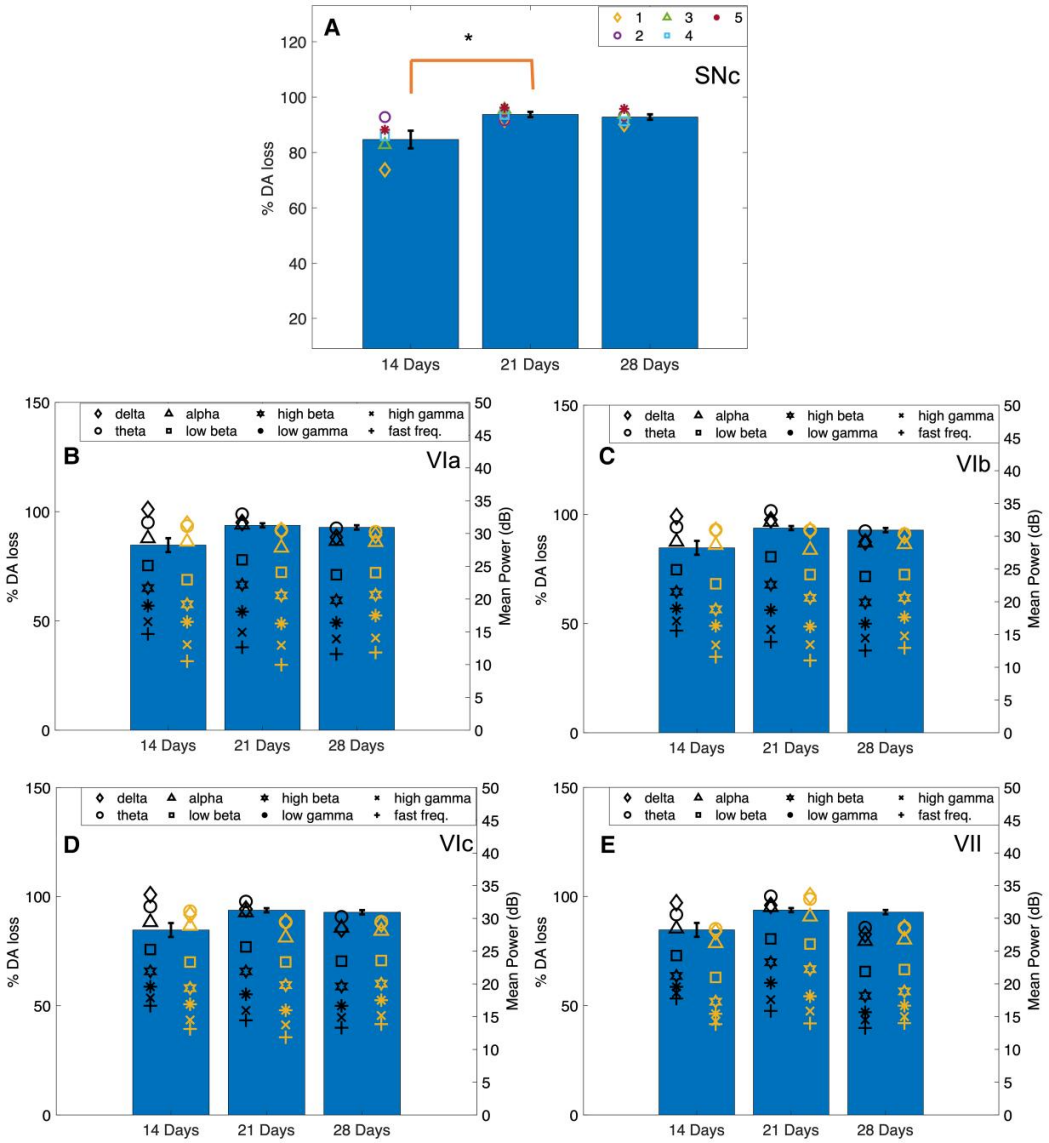
**Cell counts and LFP activity in CBLv.** Group A, Group B and 6-OHDA rats were lesioned with 6-OHDA. Subsequent DA loss was measured over time using immunohistochemistry. (**A**) Percent DA loss increased in the SNc from 14 to 21 days then remained constant from 21 to 28 days post-lesion. Each point represents the mean percent DA loss measured in each 6-OHDA rat between 14 (*n* = 5), 21 (*n* = 5) and 28 days (*n* = 5). *P < 0.05. (**B–E**) Plots displaying the effect of DA loss in the SNc on CBLv power during walking and freezing in each lobule. Each point represents the mean power in each frequency band. The symbols on the left side indicate LFP power during walking and the symbols on the right side represent LFP power during freezing at 14, 21 and 28 days. Bars indicate mean ± SEM. Comparisons were made using a one-way ANOVA. LFP, local field potential; SNc, substantia nigra pars compacta.

**Figure 7:**
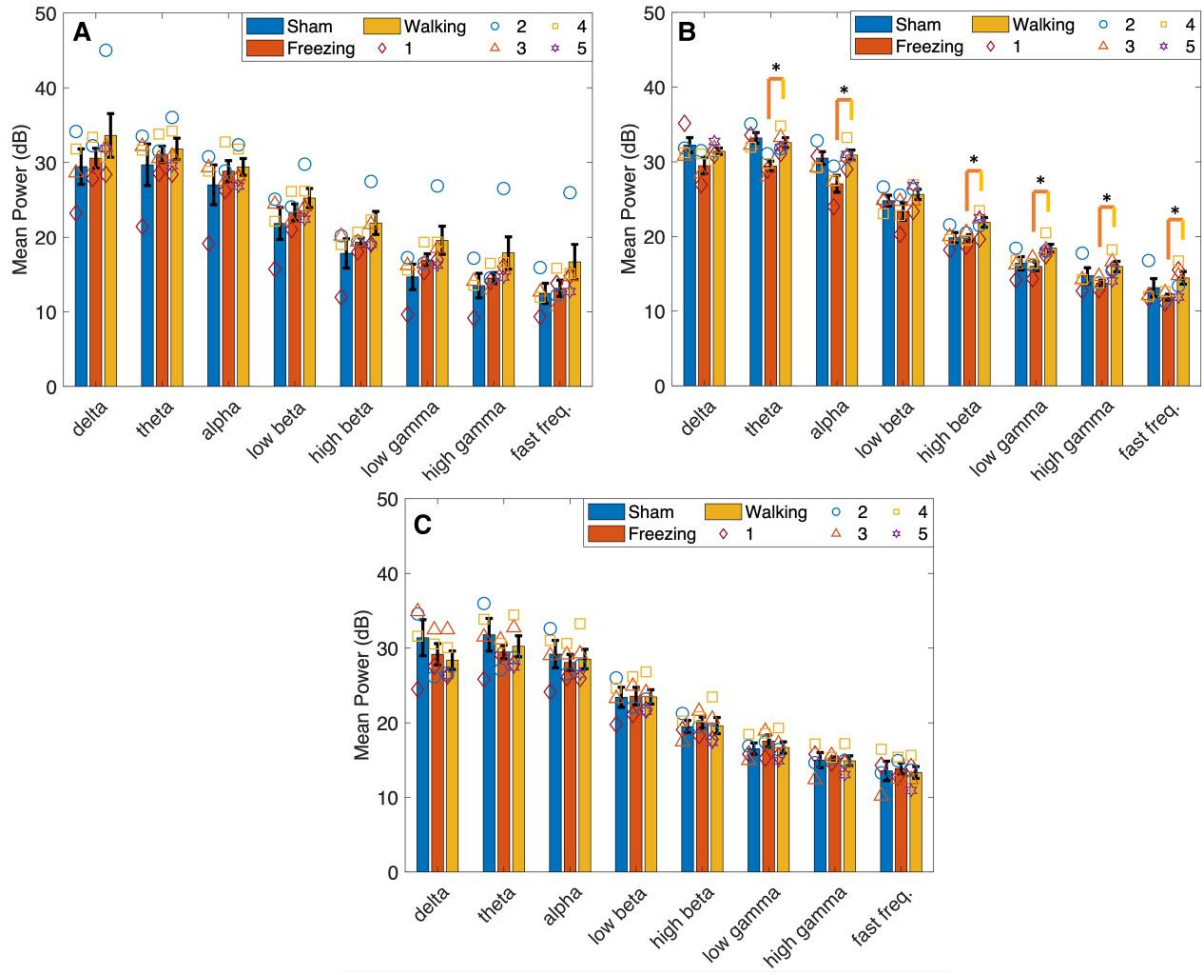
**Mean power calculated in Lobule VIc.** Cerebellar activity was measured in Lobule VIc of sham and 6-OHDA rats during walking and walking and freezing, respectively, at 14, 21 and 28 days post-lesion. (**A**) No significant differences were found in mean power [decibels (dB)] measured in sham rats during walking and 6-OHDA rats during walking and freezing 14 days after lesion surgery. (**B**) Mean power measured in sham and 6-OHDA rats at 21 days post-lesion. Hypoactivity was measured in 6-OHDA rats during freezing in the theta, alpha, high-beta, low-gamma and high-gamma, and fast frequency bands compared with walking. No significant differences were measured during walking between power in 6-OHDA and sham rats. (**C**) No significant differences in power were measured 28 days post-lesion. Symbols represent the mean at each time point in (*n* = 5) 6-OHDA rats and (*n* = 4) sham control rats. *P < 0.05. Bars indicate mean ± SEM. Comparisons were made using univariate statistical analysis.

**Figure 8:**
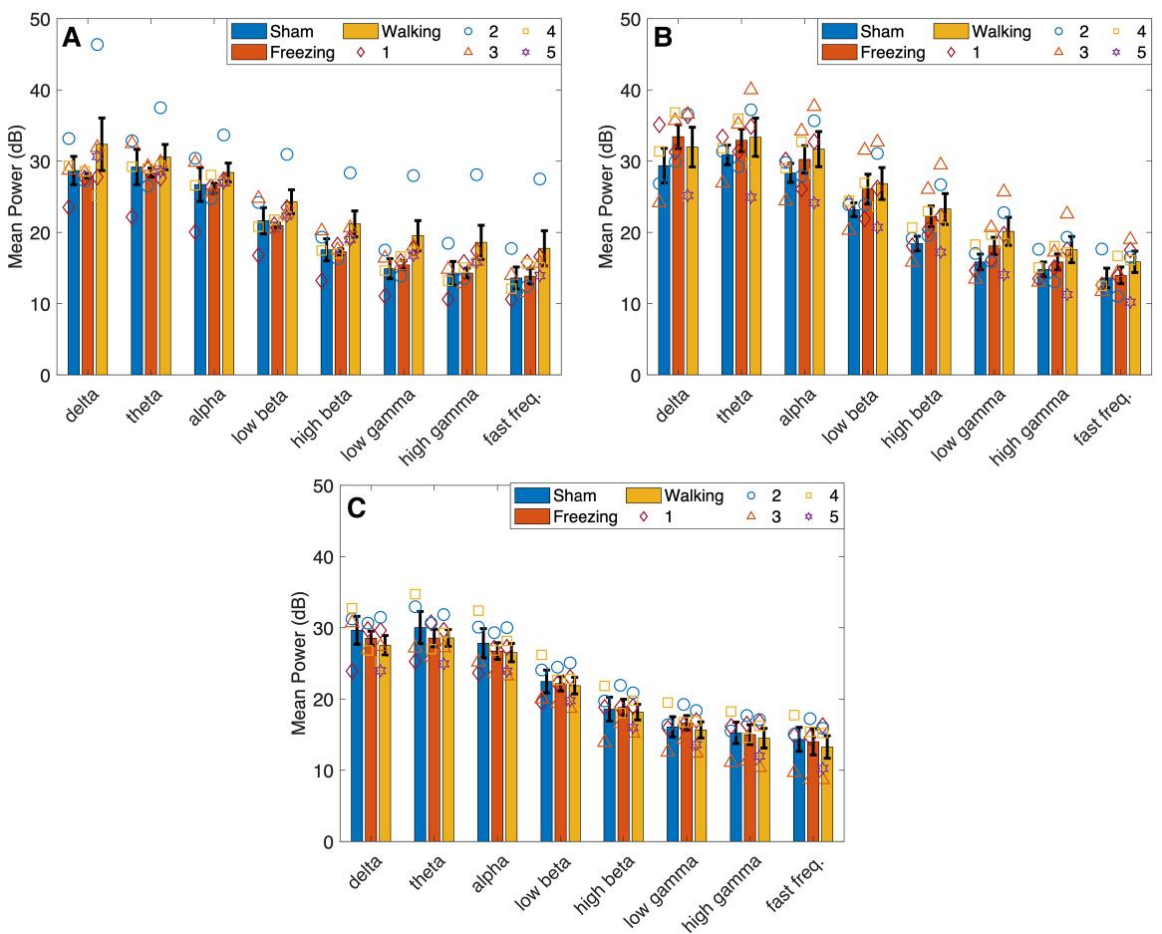
**Mean power calculated in Lobule VII.** Cerebellar activity was measured in Lobule VII of sham and 6-OHDA rats during walking and walking and freezing, respectively, at 14, 21 and 28 days post-lesion. (**A**) No significant differences were found in mean power [measured in decibels (dB)] measured in sham rats during walking and 6-OHDA rats during walking and freezing 14, (**B**) 21- and (**C**) 28 days post-lesion surgery. A significant decrease in power was measured in the theta, alpha, low-beta and high-beta band from 14 to 21 days (**A, B**) and an increase in power in the delta, theta and high beta bands from 21 to 28 days (**B, C**). These associations can also be seen in Fig. 6E. Symbols represent the mean power at each time point in (*n* = 5) 6-OHDA rats and (*n* = 4) sham control rats. Bars indicate mean ± SEM. Comparisons were made using univariate statistical analysis.

The main effect time was significant in Lobule VIb in the alpha [*F*(2,14) = 4.932, *P* = 0.024] and low-beta [*F*(2,14) = 4.102, *P* = 0.040] bands (Fig. 5). Pairwise analysis found power was significantly higher at 21 days post-lesion compared with 28 days in both frequency bands (alpha: *P* = 0.042, low beta: *P* = 0.046). *Post hoc* analysis of Lobule VIb power using a one-way repeated measures ANOVA found a significant decrease from 21 to 28 days in the alpha (*P* = 0.042) and low-gamma (*P* = 0.024) bands only in the 6-OHDA-lesioned group (Fig. 6D). No significant main effect for time or type was found in Lobules VIc (Fig. 7) and VII (Fig. 8).

#### Freezing

Fourteen days after 6-OHDA lesioning power during walking was the largest compared with freezing and sham. Power during freezing was reduced to values greater than (Lobules VIa and VIb; Figs 4 and 5) or equal to (Lobules VIc and VII; Figs 7 and 8) sham power. At 21 days, power during freezing exhibited hypoactivity in comparison with walking in both groups except in Lobule VII; however, power during freezing was still larger than power measured by the sham group during walking. At 28 days, power during walking in 6-OHDA and sham and during freezing in 6-OHDA normalized to similar levels.

No statistically significant interaction between type and time was found for any of the frequency bands in all lobules during walking. No significant main effects for time or type were found in Lobules VIa, VIb and VIc. The main effect for time was found significant in the theta [*F*(2,14) = 5.232, *P* = 0.020], alpha [*F*(2,14) = 4.776, *P* = 0.026], low beta [*F*(2,14) = 5.362, *P* = 0.019] and high beta [*F*(2,14) = 5.235, *P* = 0.020] of Lobule VII. Pairwise analysis revealed a significant decrease in power in the theta (*P* = 0.048), alpha (*P* = 0.046), low beta (*P* = 0.037) and high beta (*P* = 0.027) from 14 to 21 days, and a significant increase in power from 21 to 28 days in the delta (*P* = 0.018), theta (*P* = 0.038) and high beta (*P* = 0.048) bands (Fig. 6F). *Post hoc* analysis found no further significant differences in power during walking or freezing individually in frequency bands between time points.

Simple effect univariate analysis at each time point found significant differences in power during walking and freezing in the alpha band of Lobules VIa (*P* = 0.022), VIb (*P* = 0.006) and VIc (*P* = 0.018), the theta, high-beta, low-gamma, high-gamma and fast frequency bands of Lobules VIb (*P* = 0.014, *P* = 0.035, *P* = 0.017, *P* = 0.029 and *P* = 0.035) and VIc (*P* = 0.013, *P* = 0.018, *P* = 0.041, *P* = 0.019, *P* = 0.036 and *P* = 0.039) at 21 days post-lesion. Power during freezing was significantly reduced from the power measured during walking at this time point.

### Cell counting

Cell counting in the SNc resulted in a mean TH+ cells loss of 84.7% at 14 days (Group A), 93.7% at 21 days (Group B) and 92.7% loss at 28 days (6-OHDA) following the 6-OHDA lesion (Supplementary Fig. 2). Sham rats had a mean loss of 25% after 28 days. SNc DA loss was significant in rats lesioned with 6-OHDA between 14 and 21 days (*P* = 0.026) but not 21 and 28 days (Fig. 6A).

Figure 6B–E compares the TH+ cells DAergic cell loss in the SNc to mean power during walking and freezing at each time point. From 14 to 21 days, mean LFP power in the delta, theta and alpha frequency bands became more similar, while low-beta and high-beta power increased. This was mimicked during freezing except in Lobule VII in which power during freezing increased between time point. Power measured during freezing was lower than during walking at 14 and 21 days. At 28 days, however, this normalized where power during walking and freezing were more closely related across all lobules.

## Discussion

This study analysed the effect of DA depletion in the SNc on gait dysfunction and CBLv activity in 6-OHDA rats. A μECoG array was placed over vermis Lobules VI, VII and VII due to their involvement in the regulation of complex movements and motor planning.^26,27^ These lobules receive signals by way of the fastigial nucleus from brain regions including the motor cortex, BG and PPN, all of which are associated with gait dysfunction in Parkinson’s disease.^42^

All measurements were made at 14 days (Group A), 21 days (Group B) and 28 days (6-OHDA) following infusion with 6-OHDA and sterile saline. DAergic cell loss was over time was as follows: 84.7% at 14 days, 93.7% at 21 days and 92.7% at 28 days. To ascertain differences in cerebellar activity over time, it was important to use the same rats for gait and cerebellar recordings at all time points. Lesioning separate groups and perfusing them at three separate time points allowed us to be consistent with our measurements while still gaining an understanding of DA depletion over time. DA loss significantly increased from 14 to 21 days and plateaued from 21 to 28 days. This is consistent with what was reported by Hsieh *et al*.,^9^ although the percent DA loss reported here was greater at 21 days (93.7 versus 88.66%). The higher dosage of 6-OHDA (16 versus 8 μg) we used may explain the increased severity of the DA lesion.

Gait analysis was performed during walking in 6-OHDA- and sham-lesioned rats at 14, 21 and 28 days post-lesion. 6-OHDA gait measurements at 14 days were significantly different from sham rats and consistent with measures previously reported.^8,12^ 6-OHDA rats experienced increased stance time, stride time and swing time and decreased stride length and run speed when compared with sham controls. Gait measurements in sham rats remained consistent at all time points, whereas 6-OHDA rats exhibited an improvement in gait deficits from 14 to 21 days, despite a significant increase in DAergic cell loss. Measurements at 21 and 28 days indicated deficits still existed but were no longer significantly different from sham controls, with the exception of run speed at 28 days.

6-OHDA rats moved more quickly at 21 days than at 14 and 28 days. Run speed was the only parameter to be significantly different from sham rats at 28 days. Improvements in gait may be a result of compensatory adjustments made by the non-lesioned side during gait. Hsieh *et al*.^9^ measured asymmetries between the lesioned and non-lesioned side in 6-OHDA rats over time. Gait deficits may not have been as severe as previously reported due to the exclusion of freezing events from our analysis of walking.

CBLv activity was recorded during volitional gait on a runway. The Arduino embedded system allowed us to synchronize LFP recordings to rat activity. The sensor was triggered when the rat entered the camera view, which allowed offline synchronization of the LFP activity with periods of continuous gait and freezing. Most 6-OHDA rats did not immediately engage in continuous gait upon being placed on the runway, despite the ability to do so during training. One reason for this may be start hesitation, a symptom seen clinically in FOG.^43^

A FOG episode initiated when approaching a doorway may be overcome by shifting attention to an object away from them, thus distracting attention and resulting in a step length with the correct amplitude.^44^ If the start hesitation lasted for >5 min, the rats were placed into their home cage for a 5-min reset period. After the reset, the rats were returned to the runways and, in most cases, engaged in active gait. We postulate that placing rats in their home cage created an attention shift, resetting normal function, which allowed them to initiate gait when placed back on the runway. A limitation of this study was the inability to measure CBLv activity during this time. Future research may analyse cerebellar LFPs to see if any quantifiable changes in CBLv activity are seen during start hesitation between 6-OHDA and sham-lesioned rats.

CBLv activity was hyperactive compared with sham at 14 days in all lobules and frequency bands, with the greatest differences in the higher frequency bands. This trend in activity persisted at 21 days. At this time point, vermis Lobule VIa was significantly hyperactive compared with sham rats in the beta and gamma (low and high) bands. At 28 days, mean power in all frequency bands was reduced in all vermis lobules to similar values of sham rats.

Mean power was similar in Lobules VIa, VIb and VIc, across all time points; however, only VIa reached statistical significance between sham at 6-OHDA during walking at 21 days. Lobule VI has been shown to receive inputs from both the premotor and primary motor cortex,^27,45^ and both Lobules VI and VII are involved in cognition, emotion and motor planning.^26,27^ Cortical activity is reduced in Parkinson’s disease.^1^ Hyperactivation of this lobule may be a response to impaired behavioural and motor planning due to a disconnection of the cortico-BG circuits, resulting in impaired gait. Although gait impairments were not statistically significantly different at this time point, measures still showed deficits in the 6-OHDA rats compared with the controls.

Excessive beta activity (12–30 Hz) is a prominent feature in subthalamic nucleus (STN) recordings in patients with Parkinson’s disease. This activity is thought to contribute to the gait symptoms of Parkinson’s disease.^46-51^ As the BG and cerebellum are connected in a functional loop, oscillatory activity in the vermis is likely to be related to oscillatory activity in the STN.^29,52^ Oscillatory activity measurements of low beta and high beta in rats have produced different results from humans. Patients with FOG experienced increased low-beta activity during walking in the STN, with no significant increases in the high-beta band.^53^ 6-OHDA rats, however, exhibited increased high beta activity in the STN^52^ and in the substantia nigra pars reticulata (SNr)^54^ rats during rest and active gait on a treadmill, respectively. The difference between low- and high-beta activity during forced and volitional gait may give insight into the mechanism behind gait impairment selectivity in Parkinson’s disease. CBLv activity was increased during walking in both beta bands, which may be the result of the integration of other cortical regions besides the BG.

Gamma activity is also increased in the BG in Parkinson’s disease. An increase in gamma activity is postulated as a compensatory mechanism for the increase in beta activity but cannot fully compensate due to the inhibitory effect of beta,^55^ which could explain the gait improvements in 6-OHDA rats at 21 days. Changes in cerebellar activity from 14 to 21 days may be a response to the 10% increase in DA loss between the time points.

Cerebellar activity 28 days post-lesion may be attributed to DA loss. DA loss remained consistent from 21 to 28 days. As the CBLv receives inputs from both hemispheres of BG,^52^ this can be attributed to compensation from the non-lesioned side of the brain. Although ∼94% DA loss was measured on the lesioned side of the brain, total DA loss from both hemispheres was only about 50%. The STN is hyperactive in Parkinson’s disease^56^ but may function normally on the non-lesioned side of the brain in unilateral 6-OHDA rats. To our knowledge, STN activity in unilaterally lesioned 6-OHDA rats has only been measured ipsilateral to the lesion.^19,57^

CBLv activity decreased during freezing. These changes were measured in the alpha band of Lobule VIa and the theta, alpha, high-beta, low-gamma and high-gamma frequency bands in Lobules VIb and VIc at 21 days. Similar activity measured in Lobules VIb and VIc supports the correct placement of the μECoG arrays, because prior research has shown that these lobules project to the same brainstem nuclei.^58^ Studies measuring oscillatory activity during walking and standing/rest, have shown increased activity in the high frequency bands (high beta and gamma) and decreased activity in the low frequency (theta, alpha and low beta) bands during walking compared with standing in the SNr of 6-OHDA rats compared with non-lesioned controls.^8,54^ In humans with FOG, both low- and high-beta power were reduced during standing compared with sitting in the STN.^53^ Our results showed a similar trend in the high frequencies; however, theta and alpha band activity decreased during freezing indicating different oscillatory mechanisms from rest or standing.

Cerebellar theta oscillations are associated with the intermittent control of continuous movements while alpha oscillations are associated with attention and processing.^59^ Together, suppression of these bands may result in the inability to execute motor programmes smoothly^60^ resulting in shuffled gait and the inability to re-initiate forward gait. Differences in oscillatory trends between the CBLv and the STN may be an indicator of decoupling between the two regions.^61^ It is unclear from our measures of CBLv oscillatory activity if freezing is truly an indicator of FOG in the 6-OHDA model of Parkinson’s disease, but our results are promising. Future research measuring activity before, during and after freezing in both the vermis and STN concurrently, as well as recording from healthy rats when standing, would provide further insight. These deficits were also improved at 28 days supporting our hypothesis of compensation from the non-lesioned cerebral hemisphere. Additional compensatory mechanisms at play in the lesioned side must also be considered, including increased DA release by remaining DAergic neurons and DA receptor expression in the striatum.^62^

Research on the CBLv electrophysiological contributions to gait in Parkinson’s disease is lacking. Our results suggest that DAergic cell loss may contribute to cerebellar dysfunction through its disynaptic projections to the BG. The CBLv is hyperactive during walking and reduced during freezing. We believe the mitigation of abnormal activity at 28 days was due to compensation from the non-lesioned side of the brain, which has connections to the CBLv. Further research is needed to determine if the changes measured here are truly indicative of FOG in persons with Parkinson’s disease. Identifying changes in CBLv oscillatory activity immediately before freezing from Lobules VIb and VIc may provide a biomarker that can be used in closed-loop DBS to treat FOG. Rat models that present with bilateral DA loss may be more indicative of changes in cerebellar activity in response to DAergic cell loss.

## Supplementary Material

fcae246_Supplementary_Data

## Data Availability

Raw data were generated at Stevens Institute of Technology. Derived data supporting the findings of this study are available from the corresponding author on request.
