# Supplementary material for: Cerebellar activity in hemi-parkinsonian rats during volitional gait and freezing
Source: Brain Commun. 2024 Oct 25;6(5):fcae246. doi: 10.1093/braincomms/fcae246 (PMC11503953; doi:10.1093/braincomms/fcae246)
Supplement: fcae246_Supplementary_Data [file fcae246_supplementary_data.pdf]

Supplementary Material

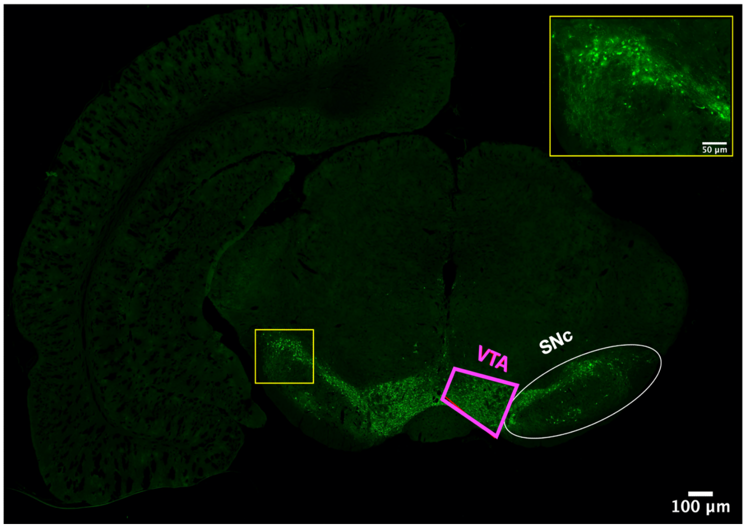

Supplementary Figure 1: Sample brain sections. Anti-TH staining to measure dopaminergic neurons in the substantia nigra pars compacta (SNc). The ventral tegmental area (VTA) was spared in 6-OHDA rats by desipramine injection prior to 6-OHDA infusion.

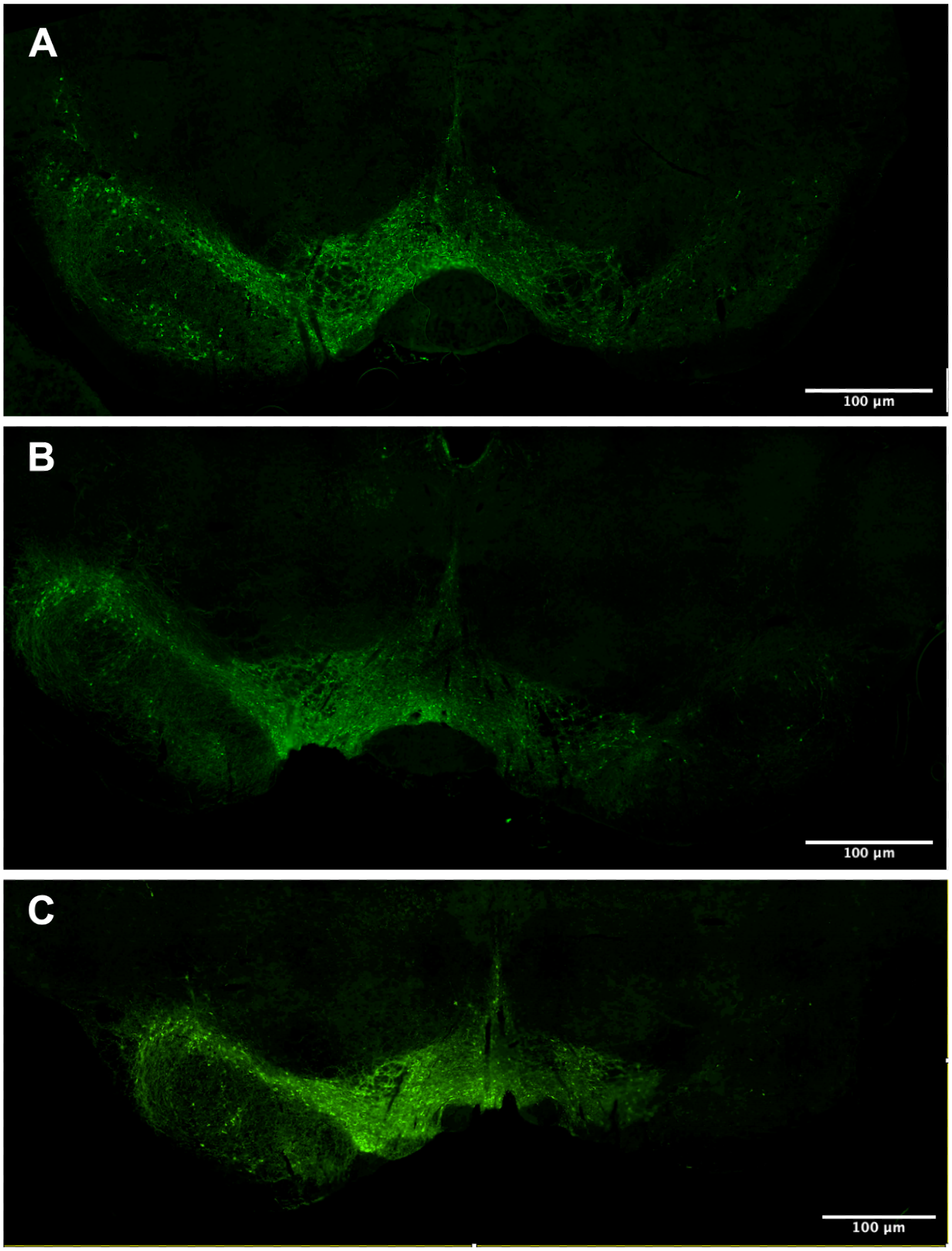

Supplementary Figure 2: Tyrosine hydroxylase immunohistochemistry. Dopaminergic TH+ cell loss in the SNc at (A) 14-, (B) 21-, and (C) 28- days after infusion of 6-OHDA in the medial forebrain bundle (MFB). Left hemisphere is non-lesioned side and right hemisphere is lesioned side for all images.

Supplementary Table 1: **Statistical analysis for rats at 21- and 28-days post lesion**. Simple effects analysis comparing gait parameters between 6-OHDA and sham rats at 21- and 28- days post lesion.

	21 days	28 days
Stride time	$F(1,7)=2.184$, $p=0.183$	$F(1,7)=4.906$, $p=0.062$
Stance time	$F(1,7)=2.044$, $p=0.196$	$F(1,7)=4.250$, $p=0.078$
Swing time	$F(1,7)=0.802$, $p=0.103$	$F(1,7)=2.160$, $p=0.185$
Stride length	$F(1,7)=2.549$, $p=0.154$	$F(1,7)=4.174$, $p=0.080$
Run speed	$F(1,7) = 0.475$, $p = 0.513$	$F(1,7) = 9.563$, $p = 0.018$
